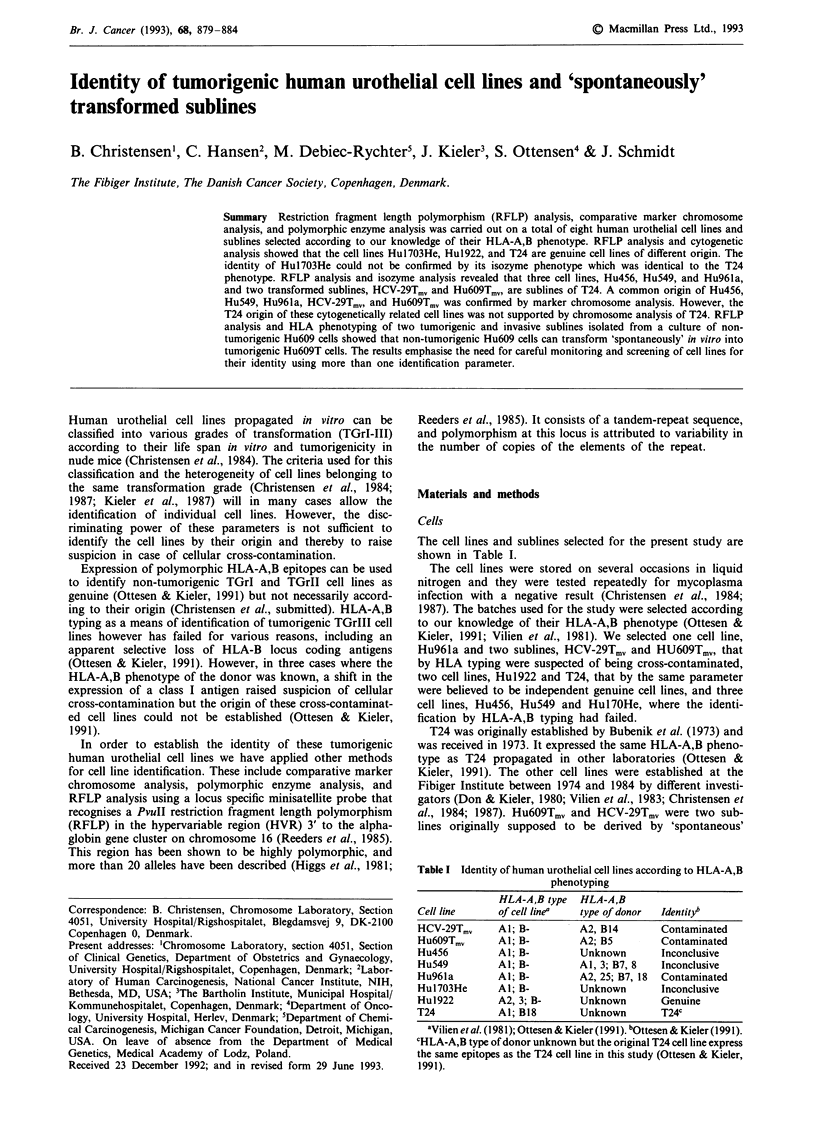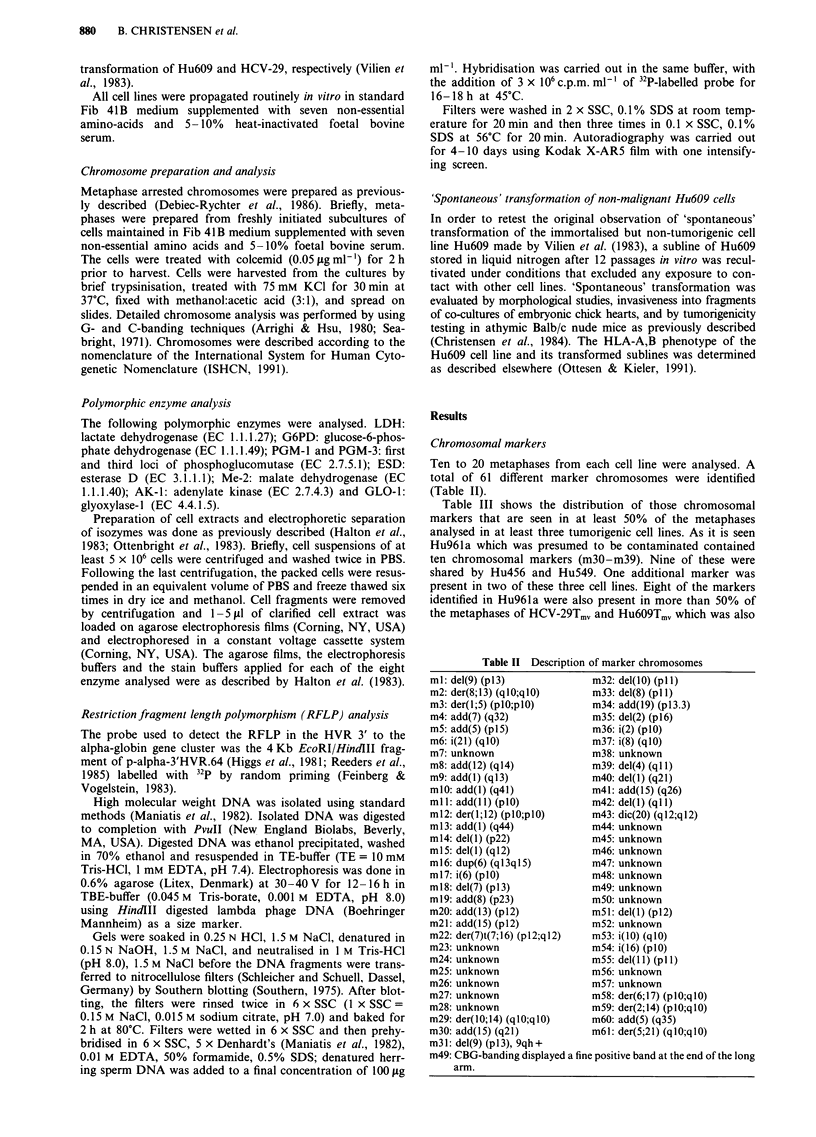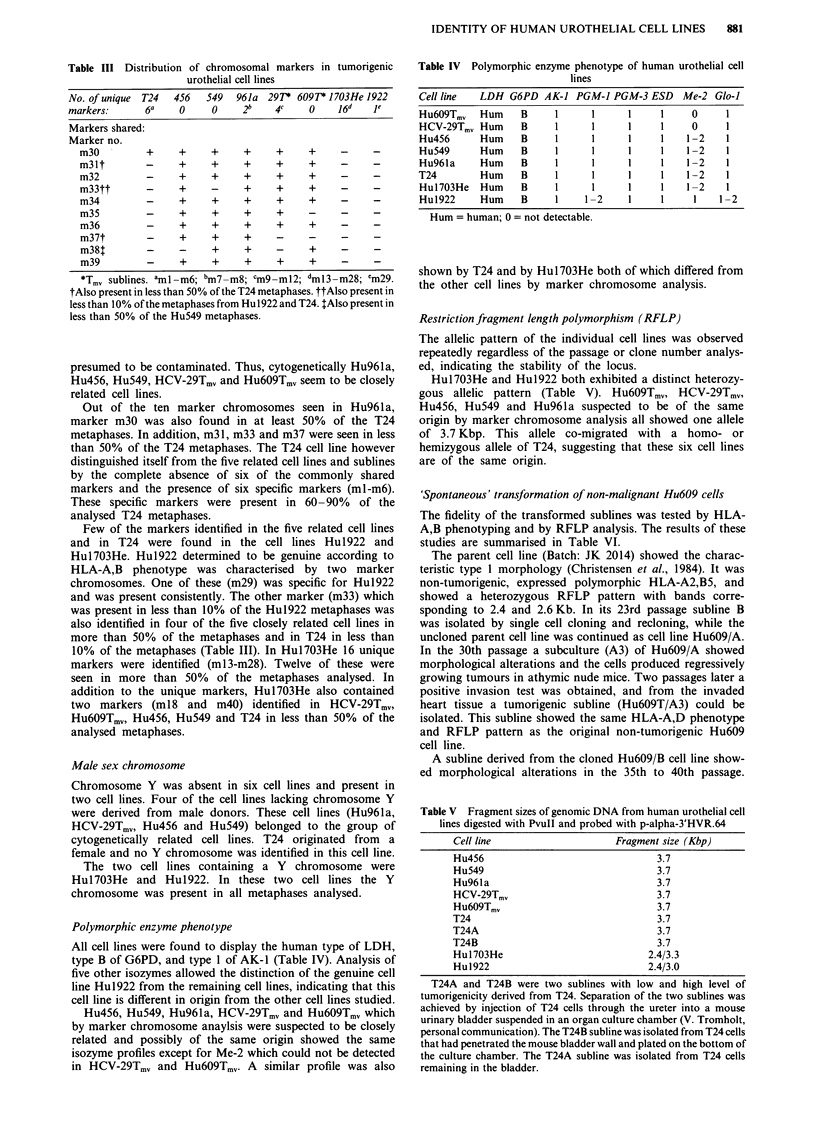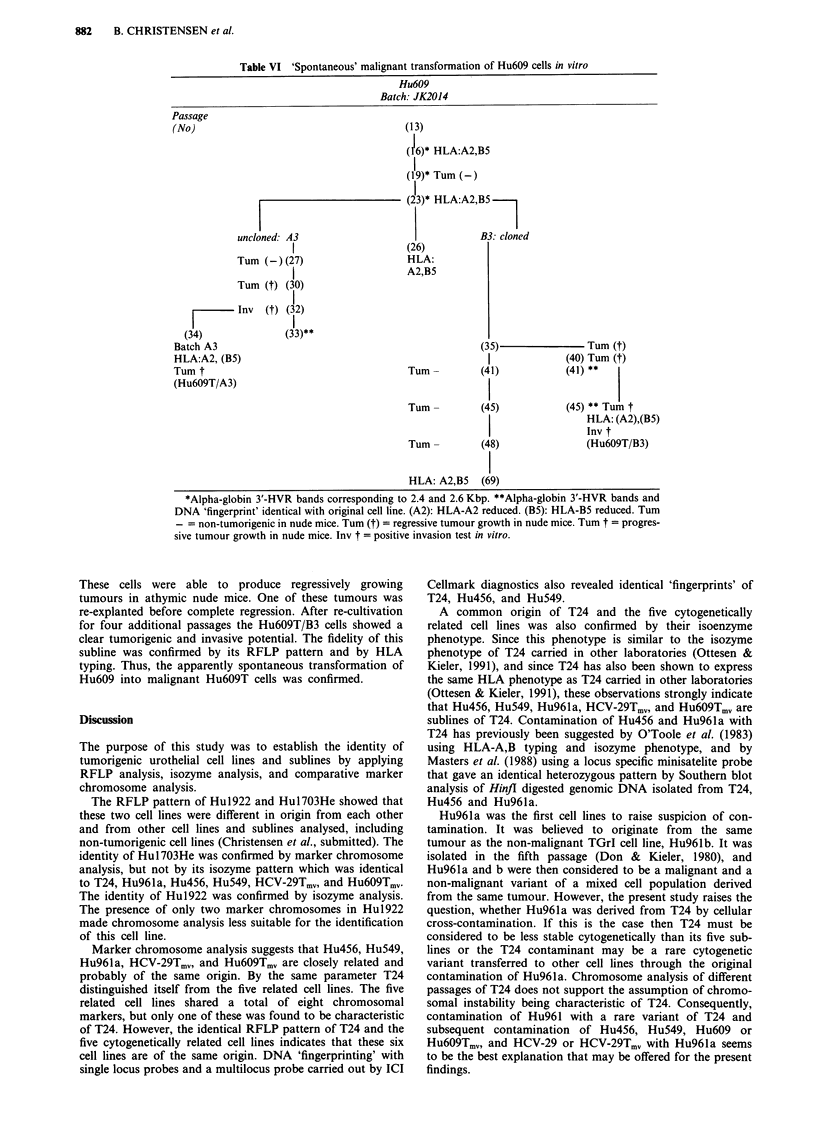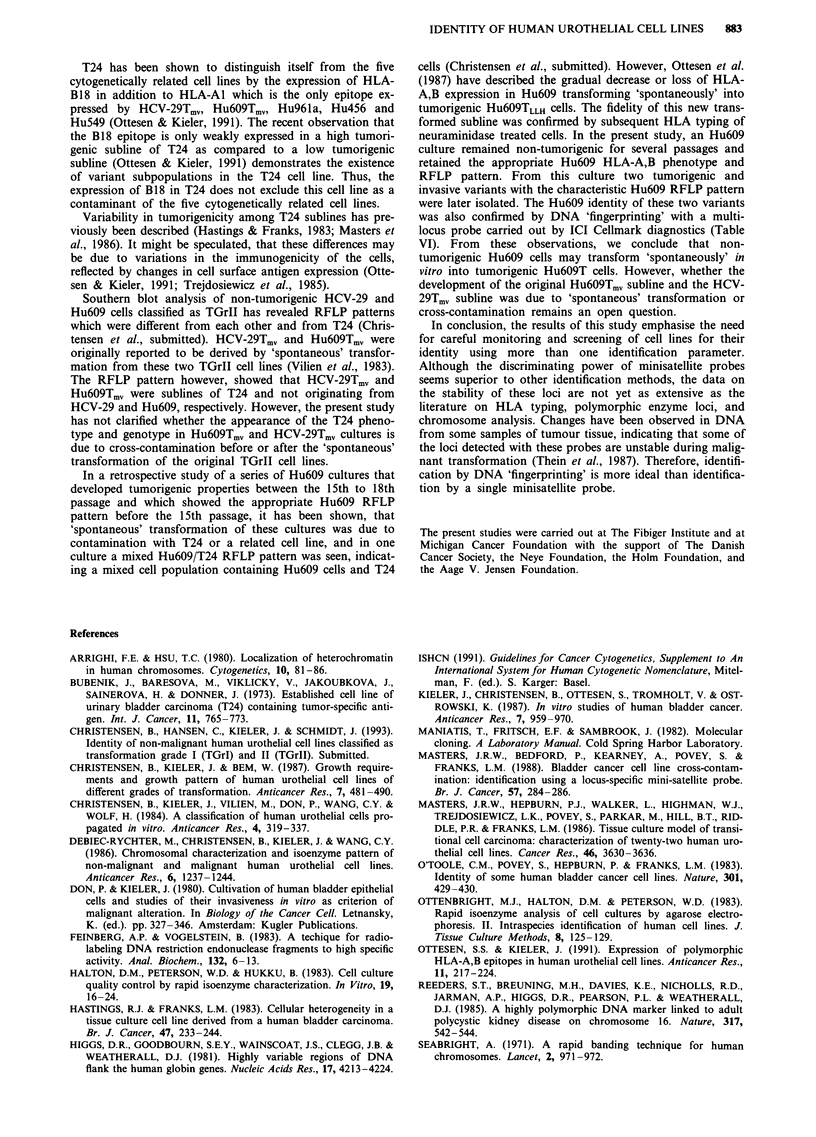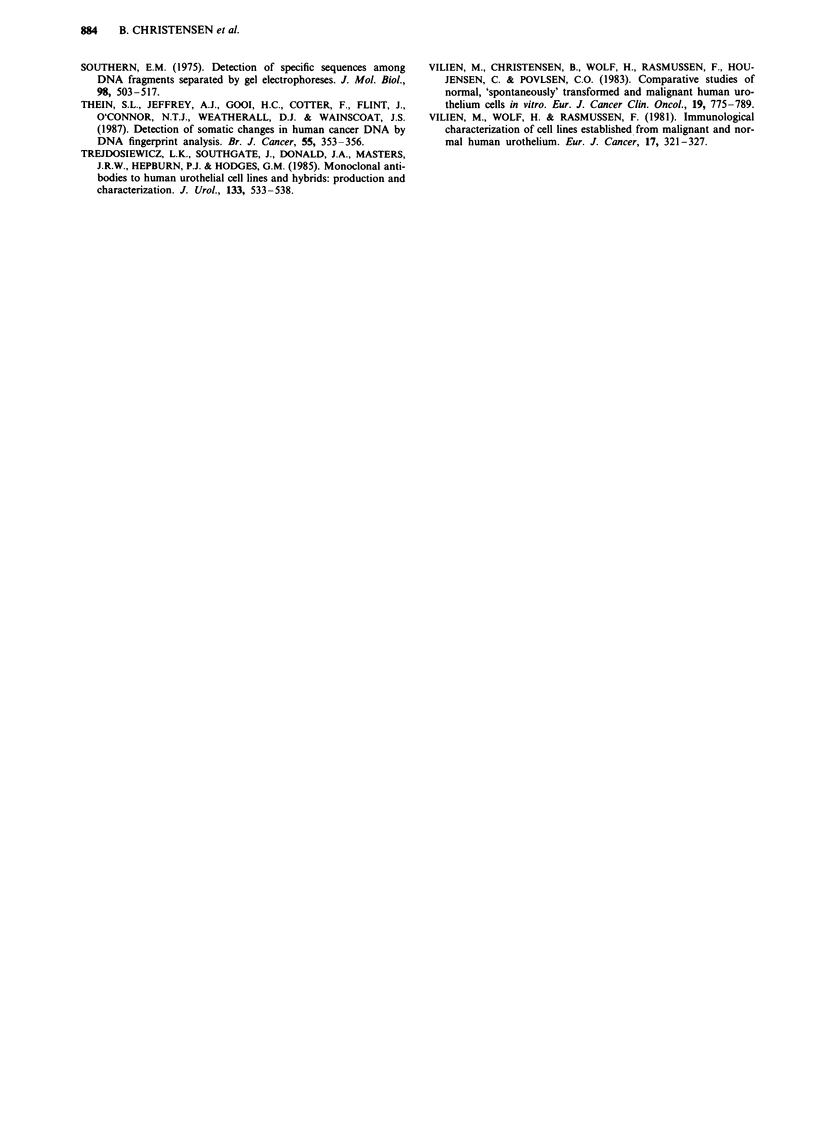# Identity of tumorigenic human urothelial cell lines and 'spontaneously' transformed sublines.

**DOI:** 10.1038/bjc.1993.449

**Published:** 1993-11

**Authors:** B. Christensen, C. Hansen, M. Debiec-Rychter, J. Kieler, S. Ottensen, J. Schmidt

**Affiliations:** Fibiger Institute, Danish Cancer Society, Copenhagen.

## Abstract

Restriction fragment length polymorphism (RFLP) analysis, comparative marker chromosome analysis, and polymorphic enzyme analysis was carried out on a total of eight human urothelial cell lines and sublines selected according to our knowledge of their HLA-A,B phenotype. RFLP analysis and cytogenetic analysis showed that the cell lines Hu1703He, Hu1922, and T24 are genuine cell lines of different origin. The identity of Hu1703He could not be confirmed by its isozyme phenotype which was identical to the T24 phenotype. RFLP analysis and isozyme analysis revealed that three cell lines, Hu456, Hu549, and Hu961a, and two transformed sublines, HCV-29Tmv and Hu609Tmv, are sublines of T24. A common origin of Hu456, Hu549, Hu961a, HCV-29Tmv, and Hu609Tmv was confirmed by marker chromosome analysis. However, the T24 origin of these cytogenetically related cell lines was not supported by chromosome analysis of T24. RFLP analysis and HLA phenotyping of two tumorigenic and invasive sublines isolated from a culture of non-tumorigenic Hu609 cells showed that non-tumorigenic Hu609 cells can transform 'spontaneously' in vitro into tumorigenic Hu609T cells. The results emphasise the need for careful monitoring and screening of cell lines for their identity using more than one identification parameter.


Br. J. Cancer (1993), 68, 879-884 © Macmillan Press Ltd., 1993

### Identity of tumorigenic human urothelial cell lines and 'spontaneously' transformed sublines

B. Christensen[1], C. Hansen[2], M. Debiec-Rychter[5], J. Kieler[3], S. Ottensen[4] & J. Schmidt

*The Fibiger Institute, The Danish Cancer Society, Copenhagen, Denmark.*

**Summary** Restriction fragment length polymorphism (RFLP) analysis, comparative marker chromosome analysis, and polymorphic enzyme analysis was carried out on a total of eight human urothelial cell lines and sublines selected according to our knowledge of their HLA-A,B phenotype. RFLP analysis and cytogenetic analysis showed that the cell lines Hul703He, Hu1922, and T24 are genuine cell lines of different origin. The identity of Hul703He could not be confirmed by its isozyme phenotype which was identical to the T24 phenotype. RFLP analysis and isozyme analysis revealed that three cell lines, Hu456, Hu549, and Hu961a, and two transformed sublines, HCV-29$T_{mv}$ and Hu609$T_{mv}$, are sublines of T24. A common origin of Hu456, Hu549, Hu961a, HCV-29$T_{mv}$, and Hu609$T_{mv}$ was confirmed by marker chromosome analysis. However, the T24 origin of these cytogenetically related cell lines was not supported by chromosome analysis of T24. RFLP analysis and HLA phenotyping of two tumorigenic and invasive sublines isolated from a culture of non-tumorigenic Hu609 cells showed that non-tumorigenic Hu609 cells can transform 'spontaneously' *in vitro* into tumorigenic Hu609T cells. The results emphasise the need for careful monitoring and screening of cell lines for their identity using more than one identification parameter.

Human urothelial cell lines propagated *in vitro* can be classified into various grades of transformation (TGrI-III) according to their life span *in vitro* and tumorigenicity in nude mice (Christensen *et al.*, 1984). The criteria used for this classification and the heterogeneity of cell lines belonging to the same transformation grade (Christensen *et al.*, 1984; 1987; Kieler *et al.*, 1987) will in many cases allow the identification of individual cell lines. However, the discriminating power of these parameters is not sufficient to identify the cell lines by their origin and thereby to raise suspicion in case of cellular cross-contamination.

Expression of polymorphic HLA-A,B epitopes can be used to identify non-tumorigenic TGrI and TGrII cell lines as genuine (Ottesen & Kieler, 1991) but not necessarily according to their origin (Christensen *et al.*, submitted). HLA-A,B typing as a means of identification of tumorigenic TGrIII cell lines however has failed for various reasons, including an apparent selective loss of HLA-B locus coding antigens (Ottesen & Kieler, 1991). However, in three cases where the HLA-A,B phenotype of the donor was known, a shift in the expression of a class I antigen raised suspicion of cellular cross-contamination but the origin of these cross-contaminated cell lines could not be established (Ottesen & Kieler, 1991).

In order to establish the identity of these tumorigenic human urothelial cell lines we have applied other methods for cell line identification. These include comparative marker chromosome analysis, polymorphic enzyme analysis, and RFLP analysis using a locus specific minisatellite probe that recognises a *Pvu*II restriction fragment length polymorphism (RFLP) in the hypervariable region (HVR) 3′ to the alpha-globin gene cluster on chromosome 16 (Reeders *et al.*, 1985). This region has been shown to be highly polymorphic, and more than 20 alleles have been described (Higgs *et al.*, 1981; Reeders *et al.*, 1985). It consists of a tandem-repeat sequence, and polymorphism at this locus is attributed to variability in the number of copies of the elements of the repeat.

### Materials and methods

### *Cells*

The cell lines and sublines selected for the present study are shown in Table I.

The cell lines were stored on several occasions in liquid nitrogen and they were tested repeatedly for mycoplasma infection with a negative result (Christensen *et al.*, 1984; 1987). The batches used for the study were selected according to our knowledge of their HLA-A,B phenotype (Ottesen & Kieler, 1991; Vilien *et al.*, 1981). We selected one cell line, Hu961a and two sublines, HCV-29$T_{mv}$ and HU609$T_{mv}$, that by HLA typing were suspected of being cross-contaminated, two cell lines, Hu1922 and T24, that by the same parameter were believed to be independent genuine cell lines, and three cell lines, Hu456, Hu549 and Hul70He, where the identification by HLA-A,B typing had failed.

T24 was originally established by Bubenik *et al.* (1973) and was received in 1973. It expressed the same HLA-A,B phenotype as T24 propagated in other laboratories (Ottesen & Kieler, 1991). The other cell lines were established at the Fibiger Institute between 1974 and 1984 by different investigators (Don & Kieler, 1980; Vilien *et al.*, 1983; Christensen *et al.*, 1984; 1987). Hu609$T_{mv}$ and HCV-29$T_{mv}$ were two sublines originally supposed to be derived by 'spontaneous'

**Table I** Identity of human urothelial cell lines according to HLA-A,B phenotyping

| *Cell line* | *HLA-A,B type of cell line*[a] | *HLA-A,B type of donor* | *Identity*[b] |
|---|---|---|---|
| HCV-29$T_{mv}$ | A1; B- | A2, B14 | Contaminated |
| Hu609$T_{mv}$ | A1; B- | A2; B5 | Contaminated |
| Hu456 | A1; B- | Unknown | Inconclusive |
| Hu549 | A1; B- | A1, 3; B7, 8 | Inconclusive |
| Hu961a | A1; B- | A2, 25; B7, 18 | Contaminated |
| Hu1703He | A1; B- | Unknown | Inconclusive |
| Hu1922 | A2, 3; B- | Unknown | Genuine |
| T24 | A1; B18 | Unknown | T24[c] |

[a]Vilien *et al.* (1981); Ottesen & Kieler (1991). [b]Ottesen & Kieler (1991). [c]HLA-A,B type of donor unknown but the original T24 cell line express the same epitopes as the T24 cell line in this study (Ottesen & Kieler, 1991).

Correspondence: B. Christensen, Chromosome Laboratory, Section 4051, University Hospital/Rigshospitalet, Blegdamsvej 9, DK-2100 Copenhagen 0, Denmark.

Present addresses: [1]Chromosome Laboratory, section 4051, Section of Clinical Genetics, Department of Obstetrics and Gynaecology, University Hospital/Rigshospitalet, Copenhagen, Denmark; [2]Laboratory of Human Carcinogenesis, National Cancer Institute, NIH, Bethesda, MD, USA; [3]The Bartholin Institute, Municipal Hospital/Kommunehospitalet, Copenhagen, Denmark; [4]Department of Oncology, University Hospital, Herlev, Denmark; [5]Department of Chemical Carcinogenesis, Michigan Cancer Foundation, Detroit, Michigan, USA. On leave of absence from the Department of Medical Genetics, Medical Academy of Lodz, Poland.

Received 23 December 1992; and in revised form 29 June 1993.

transformation of Hu609 and HCV-29, respectively (Vilien et al., 1983).

All cell lines were propagated routinely *in vitro* in standard Fib 41B medium supplemented with seven non-essential amino-acids and 5–10% heat-inactivated foetal bovine serum.

*Chromosome preparation and analysis*

Metaphase arrested chromosomes were prepared as previously described (Debiec-Rychter et al., 1986). Briefly, metaphases were prepared from freshly initiated subcultures of cells maintained in Fib 41B medium supplemented with seven non-essential amino acids and 5–10% foetal bovine serum. The cells were treated with colcemid ($0.05\,\mu g\,ml^{-1}$) for 2 h prior to harvest. Cells were harvested from the cultures by brief trypsinisation, treated with 75 mM KCl for 30 min at 37°C, fixed with methanol:acetic acid (3:1), and spread on slides. Detailed chromosome analysis was performed by using G- and C-banding techniques (Arrighi & Hsu, 1980; Seabright, 1971). Chromosomes were described according to the nomenclature of the International System for Human Cytogenetic Nomenclature (ISHCN, 1991).

*Polymorphic enzyme analysis*

The following polymorphic enzymes were analysed. LDH: lactate dehydrogenase (EC 1.1.1.27); G6PD: glucose-6-phosphate dehydrogenase (EC 1.1.1.49); PGM-1 and PGM-3: first and third loci of phosphoglucomutase (EC 2.7.5.1); ESD: esterase D (EC 3.1.1.1); Me-2: malate dehydrogenase (EC 1.1.1.40); AK-1: adenylate kinase (EC 2.7.4.3) and GLO-1: glyoxylase-1 (EC 4.4.1.5).

Preparation of cell extracts and electrophoretic separation of isozymes was done as previously described (Halton et al., 1983; Ottenbright et al., 1983). Briefly, cell suspensions of at least $5 \times 10^6$ cells were centrifuged and washed twice in PBS. Following the last centrifugation, the packed cells were resuspended in an equivalent volume of PBS and freeze thawed six times in dry ice and methanol. Cell fragments were removed by centrifugation and 1–5 µl of clarified cell extract was loaded on agarose electrophoresis films (Corning, NY, USA) and electrophoresed in a constant voltage cassette system (Corning, NY, USA). The agarose films, the electrophoresis buffers and the stain buffers applied for each of the eight enzyme analysed were as described by Halton et al. (1983).

*Restriction fragment length polymorphism (RFLP) analysis*

The probe used to detect the RFLP in the HVR 3′ to the alpha-globin gene cluster was the 4 Kb *Eco*RI/*Hind*III fragment of p-alpha-3′HVR.64 (Higgs et al., 1981; Reeders et al., 1985) labelled with $^{32}P$ by random priming (Feinberg & Vogelstein, 1983).

High molecular weight DNA was isolated using standard methods (Maniatis et al., 1982). Isolated DNA was digested to completion with *Pvu*II (New England Biolabs, Beverly, MA, USA). Digested DNA was ethanol precipitated, washed in 70% ethanol and resuspended in TE-buffer (TE = 10 mM Tris-HCl, 1 mM EDTA, pH 7.4). Electrophoresis was done in 0.6% agarose (Litex, Denmark) at 30–40 V for 12–16 h in TBE-buffer (0.045 M Tris-borate, 0.001 M EDTA, pH 8.0) using *Hind*III digested lambda phage DNA (Boehringer Mannheim) as a size marker.

Gels were soaked in 0.25 N HCl, 1.5 M NaCl, denatured in 0.15 N NaOH, 1.5 M NaCl, and neutralised in 1 M Tris-HCl (pH 8.0), 1.5 M NaCl before the DNA fragments were transferred to nitrocellulose filters (Schleicher and Schuell, Dassel, Germany) by Southern blotting (Southern, 1975). After blotting, the filters were rinsed twice in 6 × SSC (1 × SSC = 0.15 M NaCl, 0.015 M sodium citrate, pH 7.0) and baked for 2 h at 80°C. Filters were wetted in 6 × SSC and then prehybridised in 6 × SSC, 5 × Denhardt's (Maniatis et al., 1982), 0.01 M EDTA, 50% formamide, 0.5% SDS; denatured herring sperm DNA was added to a final concentration of 100 µg $ml^{-1}$. Hybridisation was carried out in the same buffer, with the addition of $3 \times 10^6$ c.p.m. $ml^{-1}$ of $^{32}P$-labelled probe for 16–18 h at 45°C.

Filters were washed in 2 × SSC, 0.1% SDS at room temperature for 20 min and then three times in 0.1 × SSC, 0.1% SDS at 56°C for 20 min. Autoradiography was carried out for 4–10 days using Kodak X-AR5 film with one intensifying screen.

*'Spontaneous' transformation of non-malignant Hu609 cells*

In order to retest the original observation of 'spontaneous' transformation of the immortalised but non-tumorigenic cell line Hu609 made by Vilien et al. (1983), a subline of Hu609 stored in liquid nitrogen after 12 passages *in vitro* was recultivated under conditions that excluded any exposure to contact with other cell lines. 'Spontaneous' transformation was evaluated by morphological studies, invasiveness into fragments of co-cultures of embryonic chick hearts, and by tumorigenicity testing in athymic Balb/c nude mice as previously described (Christensen et al., 1984). The HLA-A,B phenotype of the Hu609 cell line and its transformed sublines was determined as described elsewhere (Ottesen & Kieler, 1991).

### Results

*Chromosomal markers*

Ten to 20 metaphases from each cell line were analysed. A total of 61 different marker chromosomes were identified (Table II).

Table III shows the distribution of those chromosomal markers that are seen in at least 50% of the metaphases analysed in at least three tumorigenic cell lines. As it is seen Hu961a which was presumed to be contaminated contained ten chromosomal markers (m30–m39). Nine of these were shared by Hu456 and Hu549. One additional marker was present in two of these three cell lines. Eight of the markers identified in Hu961a were also present in more than 50% of the metaphases of HCV-29$T_{mv}$ and Hu609$T_{mv}$ which was also

**Table II** Description of marker chromosomes

| | |
|---|---|
| m1: del(9) (p13) | m32: del(10) (p11) |
| m2: der(8;13) (q10;q10) | m33: del(8) (p11) |
| m3: der(1;5) (p10;p10) | m34: add(19) (p13.3) |
| m4: add(7) (q32) | m35: del(2) (p16) |
| m5: add(5) (p15) | m36: i(2) (p10) |
| m6: i(21) (q10) | m37: i(8) (q10) |
| m7: unknown | m38: unknown |
| m8: add(12) (q14) | m39: del(4) (q11) |
| m9: add(1) (q13) | m40: del(1) (q21) |
| m10: add(1) (q41) | m41: add(15) (q26) |
| m11: add(11) (p10) | m42: del(1) (q11) |
| m12: der(1;12) (p10;p10) | m43: dic(20) (q12;q12) |
| m13: add(1) (q44) | m44: unknown |
| m14: del(1) (p22) | m45: unknown |
| m15: del(1) (q12) | m46: unknown |
| m16: dup(6) (q13q15) | m47: unknown |
| m17: i(6) (p10) | m48: unknown |
| m18: del(7) (p13) | m49: unknown |
| m19: add(8) (p23) | m50: unknown |
| m20: add(13) (p12) | m51: del(1) (p12) |
| m21: add(15) (p12) | m52: unknown |
| m22: der(7)t(7;16) (p12;q12) | m53: i(10) (q10) |
| m23: unknown | m54: i(16) (p10) |
| m24: unknown | m55: del(11) (p11) |
| m25: unknown | m56: unknown |
| m26: unknown | m57: unknown |
| m27: unknown | m58: der(6;17) (p10;q10) |
| m28: unknown | m59: der(2;14) (p10;q10) |
| m29: der(10;14) (q10;q10) | m60: add(5) (q35) |
| m30: add(15) (q21) | m61: der(5;21) (q10;q10) |
| m31: del(9) (p13), 9qh+ | |

m49: CBG-banding displayed a fine positive band at the end of the long arm.

**Table III** Distribution of chromosomal markers in tumorigenic urothelial cell lines

| *No. of unique markers:* | *T24* 6[a] | *456* 0 | *549* 0 | *961a* 2[b] | *29T** 4[c] | *609T** 0 | *1703He* 16[d] | *1922* 1[e] |
|---|---|---|---|---|---|---|---|---|
| Markers shared: | | | | | | | | |
| Marker no. | | | | | | | | |
| m30 | + | + | + | + | + | + | – | – |
| m31† | – | + | + | + | + | + | – | – |
| m32 | – | + | + | + | + | + | – | – |
| m33†† | – | + | – | + | + | + | – | – |
| m34 | – | + | + | + | + | + | – | – |
| m35 | – | + | + | + | + | – | – | – |
| m36 | – | + | + | + | + | + | – | – |
| m37† | – | + | + | + | – | – | – | – |
| m38‡ | – | – | + | + | – | + | – | – |
| m39 | – | + | + | + | + | + | – | – |

*$T_{mv}$ sublines. [a]m1–m6; [b]m7–m8; [c]m9–m12; [d]m13–m28; [e]m29. †Also present in less than 50% of the T24 metaphases. ††Also present in less than 10% of the metaphases from Hu1922 and T24. ‡Also present in less than 50% of the Hu549 metaphases.

presumed to be contaminated. Thus, cytogenetically Hu961a, Hu456, Hu549, HCV-29$T_{mv}$ and Hu609$T_{mv}$ seem to be closely related cell lines.

Out of the ten marker chromosomes seen in Hu961a, marker m30 was also found in at least 50% of the T24 metaphases. In addition, m31, m33 and m37 were seen in less than 50% of the T24 metaphases. The T24 cell line however distinguished itself from the five related cell lines and sublines by the complete absence of six of the commonly shared markers and the presence of six specific markers (m1-m6). These specific markers were present in 60–90% of the analysed T24 metaphases.

Few of the markers identified in the five related cell lines and in T24 were found in the cell lines Hu1922 and Hu1703He. Hu1922 determined to be genuine according to HLA-A,B phenotype was characterised by two marker chromosomes. One of these (m29) was specific for Hu1922 and was present consistently. The other marker (m33) which was present in less than 10% of the Hu1922 metaphases was also identified in four of the five closely related cell lines in more than 50% of the metaphases and in T24 in less than 10% of the metaphases (Table III). In Hu1703He 16 unique markers were identified (m13-m28). Twelve of these were seen in more than 50% of the metaphases analysed. In addition to the unique markers, Hu1703He also contained two markers (m18 and m40) identified in HCV-29$T_{mv}$, Hu609$T_{mv}$, Hu456, Hu549 and T24 in less than 50% of the analysed metaphases.

### *Male sex chromosome*

Chromosome Y was absent in six cell lines and present in two cell lines. Four of the cell lines lacking chromosome Y were derived from male donors. These cell lines (Hu961a, HCV-29$T_{mv}$, Hu456 and Hu549) belonged to the group of cytogenetically related cell lines. T24 originated from a female and no Y chromosome was identified in this cell line.

The two cell lines containing a Y chromosome were Hu1703He and Hu1922. In these two cell lines the Y chromosome was present in all metaphases analysed.

### *Polymorphic enzyme phenotype*

All cell lines were found to display the human type of LDH, type B of G6PD, and type 1 of AK-1 (Table IV). Analysis of five other isozymes allowed the distinction of the genuine cell line Hu1922 from the remaining cell lines, indicating that this cell line is different in origin from the other cell lines studied.

Hu456, Hu549, Hu961a, HCV-29$T_{mv}$ and Hu609$T_{mv}$ which by marker chromosome anaylsis were suspected to be closely related and possibly of the same origin showed the same isozyme profiles except for Me-2 which could not be detected in HCV-29$T_{mv}$ and Hu609$T_{mv}$. A similar profile was also shown by T24 and by Hu1703He both of which differed from the other cell lines by marker chromosome analysis.

**Table IV** Polymorphic enzyme phenotype of human urothelial cell lines

| *Cell line* | *LDH* | *G6PD* | *AK-1* | *PGM-1* | *PGM-3* | *ESD* | *Me-2* | *Glo-1* |
|---|---|---|---|---|---|---|---|---|
| Hu609$T_{mv}$ | Hum | B | 1 | 1 | 1 | 1 | 0 | 1 |
| HCV-29$T_{mv}$ | Hum | B | 1 | 1 | 1 | 1 | 0 | 1 |
| Hu456 | Hum | B | 1 | 1 | 1 | 1 | 1–2 | 1 |
| Hu549 | Hum | B | 1 | 1 | 1 | 1 | 1–2 | 1 |
| Hu961a | Hum | B | 1 | 1 | 1 | 1 | 1–2 | 1 |
| T24 | Hum | B | 1 | 1 | 1 | 1 | 1–2 | 1 |
| Hu1703He | Hum | B | 1 | 1 | 1 | 1 | 1–2 | 1 |
| Hu1922 | Hum | B | 1 | 1–2 | 1 | 1 | 1 | 1–2 |

Hum = human; 0 = not detectable.

### *Restriction fragment length polymorphism (RFLP)*

The allelic pattern of the individual cell lines was observed repeatedly regardless of the passage or clone number analysed, indicating the stability of the locus.

Hu1703He and Hu1922 both exhibited a distinct heterozygous allelic pattern (Table V). Hu609$T_{mv}$, HCV-29$T_{mv}$, Hu456, Hu549 and Hu961a suspected to be of the same origin by marker chromosome analysis all showed one allele of 3.7 Kbp. This allele co-migrated with a homo- or hemizygous allele of T24, suggesting that these six cell lines are of the same origin.

### *'Spontaneous' transformation of non-malignant Hu609 cells*

The fidelity of the transformed sublines was tested by HLA-A,B phenotyping and by RFLP analysis. The results of these studies are summarised in Table VI.

The parent cell line (Batch: JK 2014) showed the characteristic type 1 morphology (Christensen *et al.*, 1984). It was non-tumorigenic, expressed polymorphic HLA-A2,B5, and showed a heterozygous RFLP pattern with bands corresponding to 2.4 and 2.6 Kb. In its 23rd passage subline B was isolated by single cell cloning and recloning, while the uncloned parent cell line was continued as cell line Hu609/A. In the 30th passage a subculture (A3) of Hu609/A showed morphological alterations and the cells produced regressively growing tumours in athymic nude mice. Two passages later a positive invasion test was obtained, and from the invaded heart tissue a tumorigenic subline (Hu609T/A3) could be isolated. This subline showed the same HLA-A,D phenotype and RFLP pattern as the original non-tumorigenic Hu609 cell line.

A subline derived from the cloned Hu609/B cell line showed morphological alterations in the 35th to 40th passage.

**Table V** Fragment sizes of genomic DNA from human urothelial cell lines digested with PvuII and probed with p-alpha-3'HVR.64

| *Cell line* | *Fragment size (Kbp)* |
|---|---|
| Hu456 | 3.7 |
| Hu549 | 3.7 |
| Hu961a | 3.7 |
| HCV-29$T_{mv}$ | 3.7 |
| Hu609$T_{mv}$ | 3.7 |
| T24 | 3.7 |
| T24A | 3.7 |
| T24B | 3.7 |
| Hu1703He | 2.4/3.3 |
| Hu1922 | 2.4/3.0 |

T24A and T24B were two sublines with low and high level of tumorigenicity derived from T24. Separation of the two sublines was achieved by injection of T24 cells through the ureter into a mouse urinary bladder suspended in an organ culture chamber (V. Tromholt, personal communication). The T24B subline was isolated from T24 cells that had penetrated the mouse bladder wall and plated on the bottom of the culture chamber. The T24A subline was isolated from T24 cells remaining in the bladder.

**Table VI** 'Spontaneous' malignant transformation of Hu609 cells *in vitro*

*Alpha-globin 3'-HVR bands corresponding to 2.4 and 2.6 Kbp. **Alpha-globin 3'-HVR bands and DNA 'fingerprint' identical with original cell line. (A2): HLA-A2 reduced. (B5): HLA-B5 reduced. Tum – = non-tumorigenic in nude mice. Tum (†) = regressive tumour growth in nude mice. Tum † = progressive tumour growth in nude mice. Inv † = positive invasion test *in vitro*.

These cells were able to produce regressively growing tumours in athymic nude mice. One of these tumours was re-explanted before complete regression. After re-cultivation for four additional passages the Hu609T/B3 cells showed a clear tumorigenic and invasive potential. The fidelity of this subline was confirmed by its RFLP pattern and by HLA typing. Thus, the apparently spontaneous transformation of Hu609 into malignant Hu609T cells was confirmed.

### Discussion

The purpose of this study was to establish the identity of tumorigenic urothelial cell lines and sublines by applying RFLP analysis, isozyme analysis, and comparative marker chromosome analysis.

The RFLP pattern of Hu1922 and Hu1703He showed that these two cell lines were different in origin from each other and from other cell lines and sublines analysed, including non-tumorigenic cell lines (Christensen *et al.*, submitted). The identity of Hu1703He was confirmed by marker chromosome analysis, but not by its isozyme pattern which was identical to T24, Hu961a, Hu456, Hu549, HCV-29$T_{mv}$, and Hu609$T_{mv}$. The identity of Hu1922 was confirmed by isozyme analysis. The presence of only two marker chromosomes in Hu1922 made chromosome analysis less suitable for the identification of this cell line.

Marker chromosome analysis suggests that Hu456, Hu549, Hu961a, HCV-29$T_{mv}$, and Hu609$T_{mv}$ are closely related and probably of the same origin. By the same parameter T24 distinguished itself from the five related cell lines. The five related cell lines shared a total of eight chromosomal markers, but only one of these was found to be characteristic of T24. However, the identical RFLP pattern of T24 and the five cytogenetically related cell lines indicates that these six cell lines are of the same origin. DNA 'fingerprinting' with single locus probes and a multilocus probe carried out by ICI Cellmark diagnostics also revealed identical 'fingerprints' of T24, Hu456, and Hu549.

A common origin of T24 and the five cytogenetically related cell lines was also confirmed by their isoenzyme phenotype. Since this phenotype is similar to the isozyme phenotype of T24 carried in other laboratories (Ottesen & Kieler, 1991), and since T24 has also been shown to express the same HLA phenotype as T24 carried in other laboratories (Ottesen & Kieler, 1991), these observations strongly indicate that Hu456, Hu549, Hu961a, HCV-29$T_{mv}$, and Hu609$T_{mv}$ are sublines of T24. Contamination of Hu456 and Hu961a with T24 has previously been suggested by O'Toole *et al.* (1983) using HLA-A,B typing and isozyme phenotype, and by Masters *et al.* (1988) using a locus specific minisatelite probe that gave an identical heterozygous pattern by Southern blot analysis of *Hinf*I digested genomic DNA isolated from T24, Hu456 and Hu961a.

Hu961a was the first cell lines to raise suspicion of contamination. It was believed to originate from the same tumour as the non-malignant TGrI cell line, Hu961b. It was isolated in the fifth passage (Don & Kieler, 1980), and Hu961a and b were then considered to be a malignant and a non-malignant variant of a mixed cell population derived from the same tumour. However, the present study raises the question, whether Hu961a was derived from T24 by cellular cross-contamination. If this is the case then T24 must be considered to be less stable cytogenetically than its five sublines or the T24 contaminant may be a rare cytogenetic variant transferred to other cell lines through the original contamination of Hu961a. Chromosome analysis of different passages of T24 does not support the assumption of chromosomal instability being characteristic of T24. Consequently, contamination of Hu961 with a rare variant of T24 and subsequent contamination of Hu456, Hu549, Hu609 or Hu609$T_{mv}$, and HCV-29 or HCV-29$T_{mv}$ with Hu961a seems to be the best explanation that may be offered for the present findings.

T24 has been shown to distinguish itself from the five cytogenetically related cell lines by the expression of HLA-B18 in addition to HLA-A1 which is the only epitope expressed by HCV-29$T_{mv}$, Hu609$T_{mv}$, Hu961a, Hu456 and Hu549 (Ottesen & Kieler, 1991). The recent observation that the B18 epitope is only weakly expressed in a high tumorigenic subline of T24 as compared to a low tumorigenic subline (Ottesen & Kieler, 1991) demonstrates the existence of variant subpopulations in the T24 cell line. Thus, the expression of B18 in T24 does not exclude this cell line as a contaminant of the five cytogenetically related cell lines.

Variability in tumorigenicity among T24 sublines has previously been described (Hastings & Franks, 1983; Masters *et al.*, 1986). It might be speculated, that these differences may be due to variations in the immunogenicity of the cells, reflected by changes in cell surface antigen expression (Ottesen & Kieler, 1991; Trejdosiewicz *et al.*, 1985).

Southern blot analysis of non-tumorigenic HCV-29 and Hu609 cells classified as TGrII has revealed RFLP patterns which were different from each other and from T24 (Christensen *et al.*, submitted). HCV-29$T_{mv}$ and Hu609$T_{mv}$ were originally reported to be derived by 'spontaneous' transformation from these two TGrII cell lines (Vilien *et al.*, 1983). The RFLP pattern however, showed that HCV-29$T_{mv}$ and Hu609$T_{mv}$ were sublines of T24 and not originating from HCV-29 and Hu609, respectively. However, the present study has not clarified whether the appearance of the T24 phenotype and genotype in Hu609$T_{mv}$ and HCV-29$T_{mv}$ cultures is due to cross-contamination before or after the 'spontaneous' transformation of the original TGrII cell lines.

In a retrospective study of a series of Hu609 cultures that developed tumorigenic properties between the 15th to 18th passage and which showed the appropriate Hu609 RFLP pattern before the 15th passage, it has been shown, that 'spontaneous' transformation of these cultures was due to contamination with T24 or a related cell line, and in one culture a mixed Hu609/T24 RFLP pattern was seen, indicating a mixed cell population containing Hu609 cells and T24 cells (Christensen *et al.*, submitted). However, Ottesen *et al.* (1987) have described the gradual decrease or loss of HLA-A,B expression in Hu609 transforming 'spontaneously' into tumorigenic Hu609$T_{LLH}$ cells. The fidelity of this new transformed subline was confirmed by subsequent HLA typing of neuraminidase treated cells. In the present study, an Hu609 culture remained non-tumorigenic for several passages and retained the appropriate Hu609 HLA-A,B phenotype and RFLP pattern. From this culture two tumorigenic and invasive variants with the characteristic Hu609 RFLP pattern were later isolated. The Hu609 identity of these two variants was also confirmed by DNA 'fingerprinting' with a multilocus probe carried out by ICI Cellmark diagnostics (Table VI). From these observations, we conclude that non-tumorigenic Hu609 cells may transform 'spontaneously' *in vitro* into tumorigenic Hu609T cells. However, whether the development of the original Hu609$T_{mv}$ subline and the HCV-29$T_{mv}$ subline was due to 'spontaneous' transformation or cross-contamination remains an open question.

In conclusion, the results of this study emphasise the need for careful monitoring and screening of cell lines for their identity using more than one identification parameter. Although the discriminating power of minisatellite probes seems superior to other identification methods, the data on the stability of these loci are not yet as extensive as the literature on HLA typing, polymorphic enzyme loci, and chromosome analysis. Changes have been observed in DNA from some samples of tumour tissue, indicating that some of the loci detected with these probes are unstable during malignant transformation (Thein *et al.*, 1987). Therefore, identification by DNA 'fingerprinting' is more ideal than identification by a single minisatellite probe.

The present studies were carried out at The Fibiger Institute and at Michigan Cancer Foundation with the support of The Danish Cancer Society, the Neye Foundation, the Holm Foundation, and the Aage V. Jensen Foundation.

### References

ARRIGHI, F.E. & HSU, T.C. (1980). Localization of heterochromatin in human chromosomes. *Cytogenetics*, **10**, 81–86.

BUBENIK, J., BARESOVA, M., VIKLICKY, V., JAKOUBKOVA, J., SAINEROVA, H. & DONNER, J. (1973). Established cell line of urinary bladder carcinoma (T24) containing tumor-specific antigen. *Int. J. Cancer*, **11**, 765–773.

CHRISTENSEN, B., HANSEN, C., KIELER, J. & SCHMIDT, J. (1993). Identity of non-malignant human urothelial cell lines classified as transformation grade I (TGrI) and II (TGrII). Submitted.

CHRISTENSEN, B., KIELER, J. & BEM, W. (1987). Growth requirements and growth pattern of human urothelial cell lines of different grades of transformation. *Anticancer Res.*, **7**, 481–490.

CHRISTENSEN, B., KIELER, J., VILIEN, M., DON, P., WANG, C.Y. & WOLF, H. (1984). A classification of human urothelial cells propagated *in vitro*. *Anticancer Res.*, **4**, 319–337.

DEBIEC-RYCHTER, M., CHRISTENSEN, B., KIELER, J. & WANG, C.Y. (1986). Chromosomal characterization and isoenzyme pattern of non-malignant and malignant human urothelial cell lines. *Anticancer Res.*, **6**, 1237–1244.

DON, P. & KIELER, J. (1980). Cultivation of human bladder epithelial cells and studies of their invasiveness *in vitro* as criterion of malignant alteration. In *Biology of the Cancer Cell*. Letnansky, K. (ed.). pp. 327–346. Amsterdam: Kugler Publications.

FEINBERG, A.P. & VOGELSTEIN, B. (1983). A techique for radiolabeling DNA restriction endonuclease fragments to high specific activity. *Anal. Biochem.*, **132**, 6–13.

HALTON, D.M., PETERSON, W.D. & HUKKU, B. (1983). Cell culture quality control by rapid isoenzyme characterization. *In Vitro*, **19**, 16–24.

HASTINGS, R.J. & FRANKS, L.M. (1983). Cellular heterogeneity in a tissue culture cell line derived from a human bladder carcinoma. *Br. J. Cancer*, **47**, 233–244.

HIGGS, D.R., GOODBOURN, S.E.Y., WAINSCOAT, J.S., CLEGG, J.B. & WEATHERALL, D.J. (1981). Highly variable regions of DNA flank the human globin genes. *Nucleic Acids Res.*, **17**, 4213–4224.

ISHCN (1991). *Guidelines for Cancer Cytogenetics, Supplement to An International System for Human Cytogenetic Nomenclature*, Mitelman, F. (ed.). S. Karger: Basel.

KIELER, J., CHRISTENSEN, B., OTTESEN, S., TROMHOLT, V. & OSTROWSKI, K. (1987). *In vitro* studies of human bladder cancer. *Anticancer Res.*, **7**, 959–970.

MANIATIS, T., FRITSCH, E.F. & SAMBROOK, J. (1982). Molecular cloning. *A Laboratory Manual*. Cold Spring Harbor Laboratory.

MASTERS, J.R.W., BEDFORD, P., KEARNEY, A., POVEY, S. & FRANKS, L.M. (1988). Bladder cancer cell line cross-contamination: identification using a locus-specific mini-satellite probe. *Br. J. Cancer*, **57**, 284–286.

MASTERS, J.R.W., HEPBURN, P.J., WALKER, L., HIGHMAN, W.J., TREJDOSIEWICZ, L.K., POVEY, S., PARKAR, M., HILL, B.T., RIDDLE, P.R. & FRANKS, L.M. (1986). Tissue culture model of transitional cell carcinoma: characterization of twenty-two human urothelial cell lines. *Cancer Res.*, **46**, 3630–3636.

O'TOOLE, C.M., POVEY, S., HEPBURN, P. & FRANKS, L.M. (1983). Identity of some human bladder cancer cell lines. *Nature*, **301**, 429–430.

OTTENBRIGHT, M.J., HALTON, D.M. & PETERSON, W.D. (1983). Rapid isoenzyme analysis of cell cultures by agarose electrophoresis. II. Intraspecies identification of human cell lines. *J. Tissue Culture Methods*, **8**, 125–129.

OTTESEN, S.S. & KIELER, J. (1991). Expression of polymorphic HLA-A,B epitopes in human urothelial cell lines. *Anticancer Res.*, **11**, 217–224.

REEDERS, S.T., BREUNING, M.H., DAVIES, K.E., NICHOLLS, R.D., JARMAN, A.P., HIGGS, D.R., PEARSON, P.L. & WEATHERALL, D.J. (1985). A highly polymorphic DNA marker linked to adult polycystic kidney disease on chromosome 16. *Nature*, **317**, 542–544.

SEABRIGHT, A. (1971). A rapid banding technique for human chromosomes. *Lancet*, **2**, 971–972.

SOUTHERN, E.M. (1975). Detection of specific sequences among DNA fragments separated by gel electrophoreses. *J. Mol. Biol.*, **98**, 503–517.

THEIN, S.L., JEFFREY, A.J., GOOI, H.C., COTTER, F., FLINT, J., O'CONNOR, N.T.J., WEATHERALL, D.J. & WAINSCOAT, J.S. (1987). Detection of somatic changes in human cancer DNA by DNA fingerprint analysis. *Br. J. Cancer*, **55**, 353–356.

TREJDOSIEWICZ, L.K., SOUTHGATE, J., DONALD, J.A., MASTERS, J.R.W., HEPBURN, P.J. & HODGES, G.M. (1985). Monoclonal antibodies to human urothelial cell lines and hybrids: production and characterization. *J. Urol.*, **133**, 533–538.

VILIEN, M., CHRISTENSEN, B., WOLF, H., RASMUSSEN, F., HOU-JENSEN, C. & POVLSEN, C.O. (1983). Comparative studies of normal, 'spontaneously' transformed and malignant human urothelium cells *in vitro*. *Eur. J. Cancer Clin. Oncol.*, **19**, 775–789.

VILIEN, M., WOLF, H. & RASMUSSEN, F. (1981). Immunological characterization of cell lines established from malignant and normal human urothelium. *Eur. J. Cancer*, **17**, 321–327.